# Stimuli-responsive materials in organ-on-a-chip systems: from dynamic microenvironment modelling to functional readout

**DOI:** 10.3389/fbioe.2026.1957038

**Published:** 2026-09-16

**Authors:** Chunmei Xian, Yuanyuan Wu, Bingqing Xie, Huangfan Xie

**Affiliations:** 1 Department of Neurosurgery, The Affiliated Hospital, Southwest Medical University, Luzhou, China; 2 Laboratory of Neurological Diseases and Brain Function, The Affiliated Hospital, Southwest Medical University, Luzhou, China; 3 Institute of Brain Science, Southwest Medical University, Luzhou, China; 4 Sichuan Clinical Research Center for Neurosurgery, The Affiliated Hospital, Southwest Medical University, Luzhou, China

**Keywords:** adaptive biointerfaces, hydrogel matrices, organ-on-a-chip, real-time functional readout, stimuli-responsive materials

## Abstract

Organ-on-a-chip (OoC) systems provide an *in vitro* platform for modelling tissue phenotypes and functions. Compared with conventional cell culture or animal testing, they allow dynamic responses to be monitored in a setting more closely aligned with human biology. By integrating platform materials, membrane-based biointerfaces, hydrogel matrices, and readout materials, OoC systems connect structural support, tissue-tissue communication, microenvironment regulation, and real-time monitoring under controlled conditions. However, the dynamic changes that occur in living tissues are often insufficiently reproduced by commonly used materials in these components. Stimuli-responsive materials offer a way to address this gap by changing structural, transport, or readout properties in response to defined physical, chemical, or biological cues. Such responsiveness helps OoC models better reproduce evolving tissue microenvironments and improves their usefulness for biomedical testing. Unlike prior reviews that treat OoC materials or sensing components separately, this review adopts a module-based material perspective. This review summarizes recent advances, particularly those reported over the past 3 years, in the use of stimuli-responsive materials to shape microenvironmental dynamics, mediate tissue interfaces, and enable functional readout in OoC systems. By mapping these material strategies across different roles within the chip, this review connects material design with measurable biological performance and offers a material-centred perspective for future OoC design and translation.

## 1 Introduction

The *in vitro* reconstruction of organ-like systems has been pursued for decades, driven by the need to understand human physiology, model disease mechanisms, and evaluate therapeutic responses under experimentally controlled conditions. Conventional two-dimensional cultures, three-dimensional spheroids, and tissue-engineered constructs have expanded what can be studied outside the body, yet they consistently fall short of reproducing organ-level architecture, dynamic microenvironments, and coordinated tissue functions (Huskin et al., 2023). Animal models offer systemic complexity, but species differences frequently undermine their predictive value for human responses (Vashishat et al., 2024; Loewa et al., 2023). To overcome these interspecies discrepancies, researchers have turned to human-based *in vitro* models that preserve experimental control while better reflecting tissue-level physiology (Stresser et al., 2024).

Among these human-based *in vitro* models, organ-on-a-chip (OoC) systems have been developed as a leading strategy. These microengineered platforms maintain living human cells under controlled perfusion and tissue-tissue interactions, thereby supporting the reconstruction of key organ phenotypes and functions *in vitro* (Leung et al., 2022). In OoC systems, real-time sensing modules are increasingly being incorporated to monitor barrier integrity, metabolism, electrophysiology, and mechanical function during culture (Rezaei et al., 2025). Their distinctive advantage lies not in miniaturisation alone, but in the capacity to regulate flow, gradients, mechanical stimulation, tissue organisation, and longitudinal readout within a single device. These features position OoC systems as controllable, human-relevant platforms for linking microenvironmental regulation with dynamic functional assessment. Accordingly, over the past decade, OoC platforms have expanded into gut, kidney, heart, vascular, cochlear, and multi-organ configurations for disease modelling, drug evaluation, and personalised medicine (Ingber, 2022; Zhou et al., 2025).

An OoC system typically integrates four interdependent modules: the microfluidic platform, membrane based biointerfaces, hydrogel matrices, and readout components. Early OoC designs and many current devices have relied on polydimethylsiloxane (PDMS), thermoplastics, glass, porous polymer membranes, extracellular matrix (ECM) derived gels, synthetic hydrogels, and optical or electrochemical readout elements to form these functional modules (Shou et al., 2023; Salimbeigi et al., 2022; Campbell et al., 2021; Jin et al., 2020). These materials have enabled considerable progress, but their shortcomings are equally apparent: drug absorption, mechanical mismatch, limited matrix remodelling, sensor drift, and generally poor stability under dynamic culture conditions. Collectively, these issues reveal a common gap at the materials level between the dynamic behaviour of living tissues and the components used in many OoC devices, which often lack adaptive behaviour.

The persistence of these issues has motivated growing interest in stimuli responsive materials. Unlike nonadaptive materials, these materials can alter their structural, mechanical, transport, or readout properties in response to defined physical, chemical, or biological cues (Cao et al., 2023; Tevlek and Çetin, 2024). When deployed within specific chip components, such responsiveness can make platform materials more compatible with dynamic actuation, turn membranes into adaptive biointerfaces, generate hydrogels that evolve during culture, and transduce material changes into real time readouts (Soliman et al., 2024; Doryab et al., 2024). Recent applications in vascularised organoid chips, barrier models, cardiac and neural systems, and multi organ platforms further suggest that responsiveness is becoming a design principle, although its value must be judged by measurable improvements in chip level function (Quintard et al., 2024; Yang et al., 2026). Thus, stimuli responsive materials should be evaluated not only by whether they respond, but by how effectively they improve stimulus delivery, microenvironmental adaptation, and functional measurement within OoC systems.

Responsive material design is also accelerated by the broader regulatory push for new approach methodologies (NAMs), human-biology-based tools intended to complement or replace conventional animal testing. Regulatory agencies in China, the United States, and Europe have all signalled strong support: China’s NMPA has launched the Pioneer Plan for NAMs in nonclinical drug evaluation (Center for Drug Evaluation, 2026), the U.S. Food and Drug Administration has issued guidance and a roadmap that explicitly recognise OoC systems and organoids as relevant NAM tools (U.S. Food and Drug Administration, 2025), and the European Medicines Agency is advancing NAMs acceptance through context-of-use-based evaluation and early developer engagement (European Medicines Agency, 2025). Collectively, these policy shifts indicate that OoC platforms are transitioning from exploratory laboratory models to standardised, human-relevant tools for nonclinical assessment.

This review provides an overview of recent advances, particularly those reported over the past 3 years, in stimuli-responsive materials for OoC systems. The discussion is organised around four modules: platform materials, membrane biointerfaces, hydrogel matrices, and readout materials. This module-based organisation follows the main structural and functional components of OoC systems, thereby clarifying where each responsive material acts and why its function is relevant. Compared with existing reviews that discuss OoC materials, hydrogels, membranes, or biosensors separately, this review uses a functional-role framework to ask where a responsive material acts in the chip and how that response affects biological interpretation. For each module, we examine how responsive materials contribute to dynamic actuation, adaptive interfaces, matrix remodelling, or real-time monitoring. Representative original studies discussed in this review are summarized in Table 1. We distinguish direct OoC applications from OoC-related material strategies. We also discuss current limitations, including material drift, calibration difficulty, batch variability, and long-term stability under perfusion. Finally, we outline future needs in material reporting, sensing-actuation integration, device miniaturisation, and validation against tissue-level outcomes (summarized in Figure 1). By doing so, this review provides a material-centred framework for designing OoC systems for disease modelling, drug evaluation, and biomedical translation.

**TABLE 1:** Summary of stimuli-responsive material classes, response modes, evidence status, and functional applications in organ-on-a-chip systems. The table is divided into four module-specific sub-tables to distinguish direct OoC validation from OoC-related material strategies.

Material class	Main composition/biomaterial	Response mode	Representative model	Material function and biological outcome	References	Evidence status	Key evaluation need
a) Responsive platform materials							
Mechanically responsive platform materials	COC microfluidic chip with U-cup traps and serpentine channels	Architecture-guided positioning and perfusion	Vascularized organoid-on-chip	Organize organoids, hydrogel loading, and perfusion; Supported endothelial self-organization, perfusable networks, and organoid maturation	(Quintard et al., 2024)	Validated in OoC or chip-compatible model	Flow positioning accuracy; perfusion uniformity; small-molecule absorption; long-term dimensional stability
Mechanically responsive platform materials	PDMS substrate with fluorescent beads and collagen I coating	Elastic deformation with strain-field mapping	Myogenic mechanobiology chip	Deliver cyclic strain and quantify cell-experienced mechanics; Revealed strain-dependent myogenic alignment, polarization, and growth	(Zhou et al., 2026)	Validated in OoC or chip-compatible model	Strain-map accuracy; elastic recovery; PDMS absorption; coating durability under cyclic loading
Mechanically responsive platform materials	PDMS microfluidic device with porous PCL scaffold	Compliant, perfusable vascular loading	Patient-specific aortic dissection-on-chip	Transmit shear-related cues in a vascular microenvironment; Linked elevated shear stress to endothelial dysfunction, smooth muscle switching, and PIEZO1-related remodelling	(Zhao et al., 2026b)	Validated in OoC or chip-compatible model	Wall-shear calibration; scaffold biocompatibility; flow reproducibility; patient-specific geometry validation
Photo- and thermo-responsive platform materials	PEGDA-based photocurable ink (PLInk)	Local photopolymerisation	3D printed spheroid co-culture OoC	Define channels, membranes, valves, and compartment barriers; Supported endothelial sprouting, cell reorganisation, and migration across a printed vascular barrier	(Shafique et al., 2024)	OoC-related material strategy	Polymerisation completeness; leachable testing; optical dose; barrier thickness and permeability reproducibility
Photo- and thermo-responsive platform materials	pNIPAM microactuators written inside microchannels	Reversible swelling and contraction	Dynamic microfluidic cell/particle handling	Open or narrow local flow gaps; Enabled trapping and release of particles or single cells under flow	(Barwig et al., 2024)	Validated in OoC or chip-compatible model	LCST precision; swelling reversibility; cell compatibility near actuators; flow-gap calibration
Photo- and thermo-responsive platform materials	Thin gold-film optothermal surface	Light-to-heat conversion and thermo-osmotic flow	Optothermal microfluidic manipulation	Redirect local transport near the chip surface; Enabled nanoparticle trapping, sorting, and assembly by size, charge, and composition	(Chen et al., 2024)	OoC-related material strategy	Temperature mapping; photothermal specificity; nanoparticle fouling; compatibility with live-cell perfusion
Photo- and thermo-responsive platform materials	Photoactive liquid-crystal network actuator	Photoactuation of integrated microfluidic structures	On-chip fluid-control valve/pump system	Provide remote fluidic actuation; Supported light-controlled pumping or valving without bulky external actuation	(Schmidleithner et al., 2024)	Validated in OoC or chip-compatible model	Photoactuation fatigue; response time; optical dose safety; integration with cell culture media
Photo- and thermo-responsive platform materials	Iron oxide nanoparticle magnetic-hyperthermia microfluidic device	Magnetic heat generation	Thermoregulable microfluidic device	Provide remote local heating; Enabled programmable thermal regulation for microfluidic operation	(Parames-Estevez et al., 2025)	Validated in OoC or chip-compatible model	Heat confinement; magnetic-field dose; nanoparticle stability; thermal safety for adjacent compartments
Photo- and thermo-responsive platform materials	Flexible stretchable on-chip optical tweezers	Optical trapping and manipulation	High-throughput bioparticle manipulation chip	Manipulate selected particles or cells without contact; Provided stretchable, high-throughput optical control of bioparticles	(He et al., 2026)	OoC-related material strategy	Optical-force calibration; phototoxicity; stretch-induced alignment drift; throughput under perfusion
Platform and hydrogel matrix	Microfluidic organoid-on-chip platform for liver disease modelling	High-throughput dynamic culture	Human liver disease organoid-on-chip	Scale perfused organoid modelling and screening; Supported liver disease modelling and nanotherapeutic screening	(Kong et al., 2026)	Validated in OoC or chip-compatible model	Parallel perfusion uniformity; organoid viability; assay calibration; scalability for drug screening
b) Stimuli-responsive membranes and adaptive biointerfaces							
Mechanically responsive membranes	Collagen I/BME/elastin matrix-derived membrane with BS3 crosslinking	Tunable stiffness and pressure-strain response	Barrier tissue-on-chip	Couple deformation with ECM-like barrier support; Enabled independent and combined analysis of pressure and strain effects	(Newton et al., 2025)	Validated in OoC or chip-compatible model	Strain-transfer efficiency; pressure-strain calibration; ECM batch variability; permeability during deformation
Mechanically responsive membranes	Electrospun medical-grade TPU fibrous membrane	Elastic deformation and recovery	Lung alveolar *in vitro* model	Provide stretchable, fibrous, cell-supporting barrier substrate; Supported long-term alveolar epithelial culture, monolayer formation, and surfactant protein A expression	(Mitta et al., 2025)	OoC-related material strategy	Fiber and pore uniformity; elastic fatigue; epithelial adhesion; transport stability during cyclic stretch
Mechanically responsive membranes	Di-acrylated Pluronic F127 hydrogel membrane	Curved hydrogel deformation	Curved lung barrier-on-chip	Recreate sac-like deformable tissue barrier geometry; Supported epithelial-endothelial culture under stretching and flow	(She et al., 2023)	Validated in OoC or chip-compatible model	Curved-membrane reproducibility; stretch durability; hydrogel swelling; barrier leakage under flow
Biochemical and cell mediated remodellable membranes	Ultrathin collagen I or collagen/BME ECM membrane	Adjustable thickness and permeability	Thermoplastic OoC barrier device	Replace permanent synthetic inserts with ECM-like adhesive interface; Supported epithelial and endothelial culture with closer tissue-tissue communication	(Newton et al., 2024)	OoC-related material strategy	Thickness control; permeability tuning; ECM composition reporting; stability during long-term perfusion
Biochemical and cell mediated remodellable membranes	Hydrolysed and EDC/NHS-activated PETE membrane with protein coatings	Stable protein immobilisation	Multilayer OoC membrane platform	Improve adhesion on otherwise bioinert PETE; Enhanced A549 epithelial and EA.hy926 endothelial attachment while preserving membrane integrity	(Sobejano de la Merced et al., 2025)	OoC-related material strategy	Coating density; protein retention; surface chemistry stability; cell-type-specific adhesion response
Biochemical and cell mediated remodellable membranes	Electrospun silk fibroin ultrathin membrane	ECM-like fibrous support	Human kidney glomerulus-on-chip	Support close epithelial-endothelial communication and selective filtration; Enabled podocyte-endothelial organisation, fenestrated endothelium, and disease-relevant barrier dysfunction	(Mou et al., 2024)	Validated in OoC or chip-compatible model	Selective filtration; fibrous membrane integrity; podocyte-endothelial compatibility; disease-readout validation
Membrane biointerface and impedance readout	Porous membrane with cell-substrate impedance electrodes	Impedance-based barrier readout	Microfluidic barrier-on-chip	Combine membrane culture with barrier integrity sensing; Enabled integrated monitoring of barrier function on chip	(Ugodnikov et al., 2025)	Validated in OoC or chip-compatible model	Impedance baseline drift; pore-electrode alignment; barrier calibration; medium-related electrode stability
Membrane biointerface	Porous silicon biomembrane	Defined porous transport	OoC membrane technology platform	Provide a silicon-based porous barrier substrate; Demonstrated a biomembrane fabrication route for OoC applications	(Williams et al., 2025)	OoC-related material strategy	Pore-size reproducibility; silicon biocompatibility; transport calibration; fragility during device integration
c) Stimuli-responsive hydrogel matrices							
Physicochemically responsive hydrogel networks	Sodium carboxymethyl cellulose/PVA/agarose hydrogel loaded with 5-fluorouracil	pH-dependent diffusion-controlled drug release	Renal tumour hydrogel-on-chip with A498 cells	Link matrix transport with local drug exposure; Produced sustained cytotoxic and apoptosis-related responses under dynamic culture	(Gupta et al., 2026)	Validated in OoC or chip-compatible model	pH-release profile; drug diffusion modelling; hydrogel swelling; tumour-cell viability controls
Physicochemically responsive hydrogel networks	Four-arm PEG-vinyl sulfone, hyaluronic acid, dextran, RGD, and MMP-sensitive crosslinker	Enzymatic degradation in microporous network	Perfused bone-cell network chip	Permit protease-driven spreading and matrix remodelling; Supported rapid 3D cell-network formation, long-term maintenance, and collagen deposition	(Zauchner et al., 2024)	Validated in OoC or chip-compatible model	MMP sensitivity; pore interconnectivity; degradation products; perfusion-compatible matrix remodelling
Physicochemically responsive hydrogel networks	Strained alkyne-modified HA and photosensitive azide crosslinker	Photo-softening and photo-degradation	3D cell culture matrix	Change local matrix mechanics after encapsulation; Promoted cell spreading and primitive capillary-like network formation in irradiated regions	(Palmieri et al., 2025)	OoC-related material strategy	Light-dose response; softening reversibility; phototoxicity; spatial resolution of matrix change
ECM mimicking and cell mediated remodelling hydrogel matrices	Gelatin/beta-cyclodextrin host-guest hydrogel	Rapid stress relaxation	Human blood vessel organoids	Provide a dynamic matrix for vascular organoid maturation; Enhanced angiogenesis and arteriole-like differentiation through Notch3- and PDGFB-related signalling	(Sun et al., 2025)	OoC-related material strategy	Stress-relaxation kinetics; host-guest stability; organoid maturation markers; batch-to-batch gelatin variability
ECM mimicking and cell mediated remodelling hydrogel matrices	Collagen VI-enriched decellularised amniotic membrane ECM hydrogel	Tissue-derived biochemical support	iPSC-derived islet organoids	Provide islet-relevant ECM cues; Improved organoid viability, functional maturity, biomimetic architecture, and engraftment	(Zhu et al., 2025)	OoC-related material strategy	Collagen VI content; dECM batch variability; islet functional assays; implantation versus chip relevance
ECM mimicking and cell mediated remodelling hydrogel matrices	Boronate ester dynamic hydrogel	Dynamic matrix remodelling	3D organoid culture	Balance structural support with organoid-driven reorganisation; Allowed encapsulated organoids to expand and reorganise their microenvironment	(Neumann et al., 2026)	Organoid culture platform	Dynamic bond lifetime; organoid expansion space; gel stability; reproducibility of remodelling response
ECM mimicking and cell mediated remodelling hydrogel matrices	Mechanically and chemically defined PEG hydrogel	Defined matrix remodelling	Human cardioid development	Improve reproducibility of organoid matrix conditions; Enhanced control over cardioid development compared with less-defined matrices	(Song et al., 2025)	Organoid culture platform	Matrix definition; stiffness tuning; developmental reproducibility; comparison with natural matrix controls
ECM mimicking and cell mediated remodelling hydrogel matrices	Adaptive cell-matrix hydrogel for cartilaginous organoids	Adaptive matrix response	Cartilaginous organoids	Recapitulate hypoxic matrix microenvironment; Enhanced histone lactylation and cartilage-regeneration-related outcomes	(Yang et al., 2025)	Organoid culture platform	Hypoxia-matrix coupling; degradation/remodelling kinetics; lactylation readout specificity; long-term cartilage phenotype
d) Readout-oriented responsive materials							
Electroactive and electrochemical readout materials	3D printed electrochemical multiwell plate	Electrochemical detection	Intestinal organoid food-intolerance model	Track secreted signals during organoid response; Enabled electrochemical monitoring of food-intolerance-related organoid activity	(Brooks et al., 2023)	OoC-related material strategy	Electrode-to-well reproducibility; secretion calibration; medium fouling; compatibility with organoid culture duration
Electroactive and electrochemical readout materials	Graphene field-effect transistor	Field-effect conductance modulation	Organoid drug-evaluation platform	Monitor organoid fate state in real time; Enabled non-destructive tracking of drug responses	(Tian et al., 2024)	OoC-related material strategy	Graphene functional stability; drift correction; drug-response calibration; non-destructive readout validation
Electroactive and electrochemical readout materials	Au nanotube/PDMS stretchable electrochemical interface	Electrochemical oxidation signal	Stretchable intestinal epithelium chip	Detect serotonin while maintaining a deformable culture surface; Monitored fatty-acid-enhanced serotonin release from Caco-2 epithelium	(Hong et al., 2025)	Validated in OoC or chip-compatible model	Serotonin selectivity; stretch-cycle durability; AuNT adhesion; calibration during peristalsis-like deformation
Electroactive and electrochemical readout materials	MNP@ZIF-8/enzyme sensor	Enzyme-selective electrochemical detection	hiPSC-derived neural disease-modelling platform	Convert neurotransmitter release into an *in situ* electrochemical signal; Enabled direct glutamate monitoring during neuronal culture	(Feng et al., 2026)	OoC-related material strategy	Enzyme stability; glutamate selectivity; nanomaterial biocompatibility; calibration in neuronal culture medium
Electroactive and electrochemical readout materials	Microelectrode array-integrated Transwell	Spatial TEER measurement	Cell barrier monitoring platform	Map barrier integrity across culture area; Enabled real-time spatial monitoring of epithelial barrier formation and disruption	(Shi et al., 2025)	OoC-related material strategy	Spatial TEER calibration; electrode fouling; barrier model reproducibility; comparison with conventional TEER
Electroactive and electrochemical readout materials	Kirigami electronic interface	Long-term electrical recording	Human neural organoids and assembloids	Maintain conformal contact with growing 3D neural tissues; Supported long-term electrophysiological recording of organoids and assembloids	(Yang et al., 2024)	OoC-related material strategy	Conformal contact stability; long-term recording drift; tissue growth accommodation; neural signal validation
Electroactive and electrochemical readout materials	Soft 3D bioelectrodes	Bioelectrical signal recording	Cardiac organoid model	Improve internal tissue contact for signal monitoring; Enabled intraorganoid electrical monitoring in cardiac models	(Park et al., 2025)	OoC-related material strategy	Electrode softness; intraorganoid positioning; signal-to-noise ratio; tissue damage during integration
Optical and photonic readout materials	Photonic crystal hydrogel with ordered photonic lattice	Swelling-induced wavelength shift	Microfluidic *in situ* chemical sensing	Provide local optical sensing inside channels; Converted chemical variation into spatially resolved optical signals	(Liu et al., 2024)	Validated in OoC or chip-compatible model	Wavelength calibration; swelling reversibility; ionic/pH specificity; optical stability under perfusion
Optical and photonic readout materials	Aptamer-functionalised cholesteric liquid crystal IPN film	Concentration-dependent optical shift	Photonic biosensor	Provide molecule-selective optical detection; Detected microcystin through aptamer recognition and photonic signal conversion	(Hussain et al., 2024)	OoC-related material strategy	Aptamer specificity; LC alignment stability; concentration-response range; matrix interference testing
Optical and photonic readout materials	Calcium-sensitive fluorescent indicators	Fluorescence intensity change	Imaging-compatible cardiac organoid platform	Monitor excitation-related cellular activity during pacing; Captured calcium transients and drug-related cardiac functional responses	(Tirgar et al., 2025)	OoC-related material strategy	Photobleaching; dye retention; pacing compatibility; correlation with electrophysiological readouts
Optical and photonic readout materials	Organelle-targeting two-photon activatable fluorophores	Photoactivated fluorescence	Living cell and tissue-level imaging	Enable deeper 3D subcellular visualisation; Provided a route for monitoring cellular states in thicker organoid-on-chip tissues	(Ma et al., 2025)	OoC-related material strategy	Two-photon dose; organelle specificity; tissue penetration depth; translation to integrated OoC sensors
Optical and photonic readout materials	Porous silicon optoporation material	Spatially resolved transfection	Organoid transfection platform	Enable regional manipulation for optical analysis; Supported spatially resolved organoid transfection	(Spiteri et al., 2024)	OoC-related material strategy	Transfection spatial resolution; silicon biocompatibility; optical dose; organoid viability after optoporation
Mechanical and electromechanical readout materials	Hydrogel strain sensor	Strain-dependent signal change	Dynamic organ-on-a-chip integration	Measure deformation inside dynamic OoC systems; Provided strain readout for mechanically active chip environments	(She et al., 2025)	Validated in OoC or chip-compatible model	Gauge factor; hydrogel fatigue; strain calibration under flow; coupling to deforming chip structures
Mechanical and electromechanical readout materials	Silicon nitride AFM cantilever	Cantilever deflection and torsion	Dual cardiac organoid biosensor	Convert contractile motion into biomechanical signals; Detected synchronized beating, arrhythmic conduction, and drug-induced cardiotoxicity	(Svěrák et al., 2026)	OoC-related biosensor	Cantilever sensitivity; force calibration; organoid contact stability; throughput limitation
Mechanical and electromechanical readout materials	Resistive skin coupled with microelectrode array	Resistance change and electrical recording	Dynamically beating human cardiac organoids	Profile mechanical and electrophysiological activity together; Linked contraction strength and rhythm with electrophysiological behaviour	(Yin et al., 2025)	OoC-related material strategy	Mechanical-electrical signal alignment; resistance drift; MEA coupling; contraction and rhythm calibration
Mechanical and electromechanical readout materials	Piezoresistive silicon cantilevers coupled to a microscale liquid membrane	Pressure-mediated cantilever bending and resistance change	Wireless biomechanical well plate	Measure contraction without clamping tissue or immersing sensors; Enabled submillinewton force monitoring, stimulation studies, temperature testing, and drug analysis	(Nguyen et al., 2026)	OoC-related material strategy	Pressure-force calibration; wireless drift; liquid-membrane stability; parallel-well reproducibility
Mechanical and electromechanical readout materials	Hydrogel-actuated 3D microelectrode array	3D electrode repositioning and electrical recording	Brain spheroid electrophysiology platform	Improve 3D contact between electrodes and neural tissue; Enabled electrophysiological monitoring in brain spheroid models	(Martinelli et al., 2024)	OoC-related material strategy	Electrode repositioning accuracy; hydrogel actuation stability; neural contact safety; long-term recording quality

AFM, atomic force microscopy; BME, basement membrane extract; COC, cyclic olefin copolymer; ECM, extracellular matrix; LCST, lower critical solution temperature; MMP, matrix metalloproteinase; OoC, organ-on-a-chip; PCL, polycaprolactone; PDMS, polydimethylsiloxane; PEGDA, poly (ethylene glycol) diacrylate; PETE, polyethylene terephthalate; PMMA, poly (methyl methacrylate); pNIPAM, poly (N-isopropylacrylamide); TPU, thermoplastic polyurethane.

**FIGURE 1:**
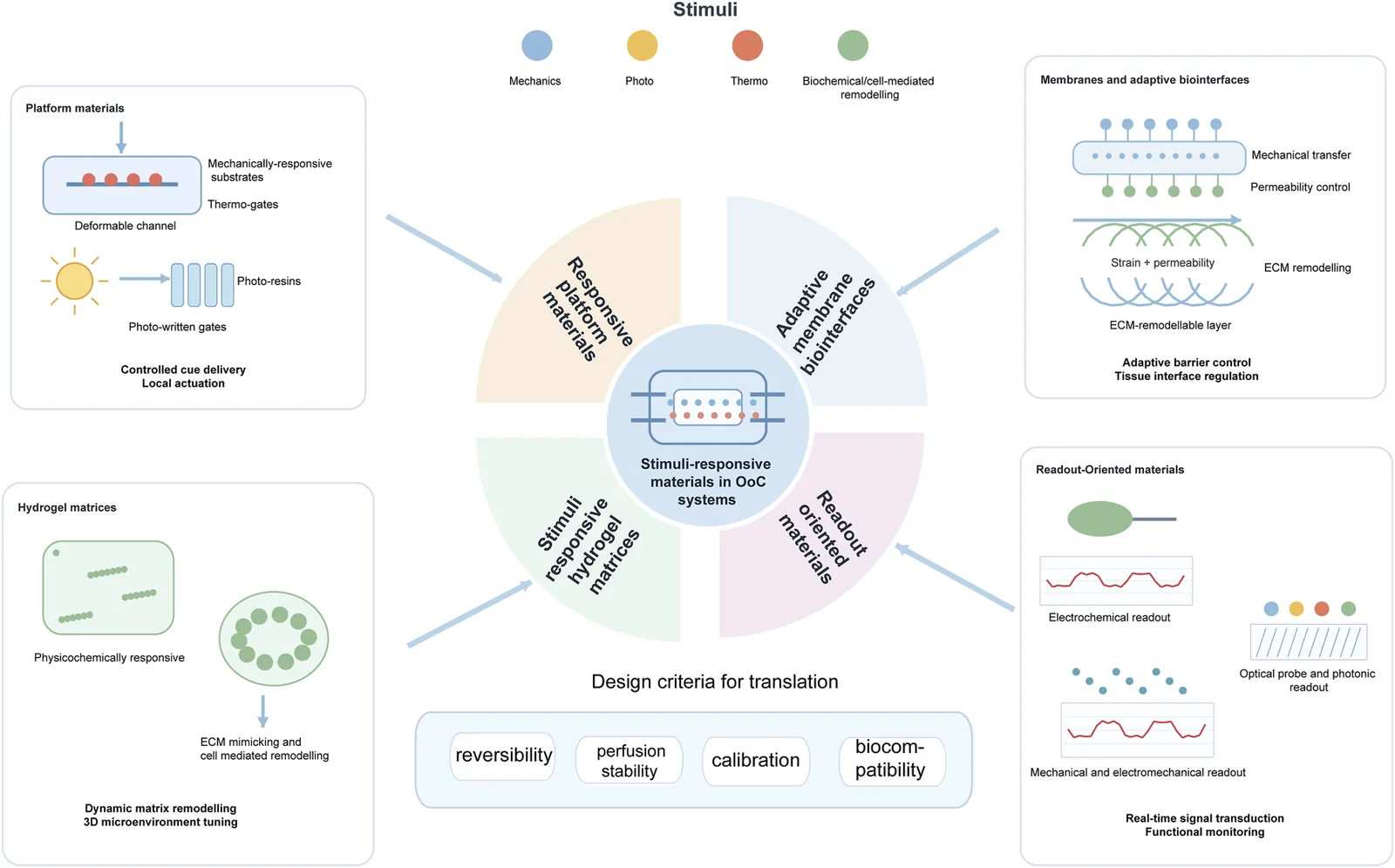
Schematic diagram of stimuli-responsive materials in organ-on-a-chip systems. The diagram summarizes four major OoC modules involving stimuli-responsive materials, including platform materials, membrane biointerfaces, hydrogel matrices, and readout materials. These materials respond to mechanical, optical, thermal, biochemical, cellular, electrical, or chemical cues and contribute to dynamic actuation, adaptive barrier interfaces, matrix remodelling, and real-time functional readout in OoC models.

## 2 Responsive platform materials

In this section, platform materials refer to the structural materials that form the microfluidic body of an OoC device, including substrates, channel walls, deformable chambers, and actuation membranes (Farhang Doost and Srivastava, 2024; Obeid et al., 2024). The term responsive platform material is used in a functional sense: it includes materials that directly change their properties after stimulation as well as structural materials whose geometry, compliance, or integration enables dynamic flow, loading, or spatial control within the chip. Under this definition, platform materials are not treated as passive supports; instead, they shape how mechanical, optical, thermal, and chemical cues are delivered, transmitted, and measured. A responsive hydrogel, membrane, or sensor may be carefully designed, but its behaviour can still be altered by the surrounding platform material. This section focuses on mechanical actuation and optical or thermal spatial control because these are the platform-level responses most directly linked to device-forming structures. Chemical and biological responsiveness are discussed mainly in the membrane, hydrogel, and readout sections, where the responsive element is usually located at the tissue interface, within the matrix, or in the sensing layer rather than in the bulk chip body (Newton et al., 2024; Soliman et al., 2024; Rezaei et al., 2025).

### 2.1 Mechanically responsive platform materials

Mechanically responsive platform materials are used when an OoC model needs to reproduce force, deformation, pressure, or tissue-generated contraction. This requirement is especially clear in vascular, cardiac, skeletal muscle, and intestinal chips, where tissue function depends on how cells sense and respond to mechanical cues, not only on how the tissue looks (Leung et al., 2022; Ingber, 2022). To deliver these cues reliably, the microfluidic structure, including channel walls, deformable chambers, posts, and supporting elements, must be able to deform, recover, and transmit force in a controlled manner (Doryab et al., 2024; Leung et al., 2022). These functions depend directly on the choice of platform material and on how that material is incorporated into the chip architecture.

The main material systems include PDMS, thermoplastic elastomers, polyurethane based elastomers, photocurable elastic resins, printed soft supports, and hybrid structures combining soft and rigid regions. Across these systems, the key design requirement is to balance deformability, recovery, bonding stability, and chemical compatibility so that mechanical inputs can be transmitted without introducing uncontrolled drift or material related artefacts (Cao et al., 2023; Campbell et al., 2021). In practice, platform level mechanical responsiveness can arise from two routes: direct material deformation and architecture defined control of flow, loading, and tissue positioning. In a vascularized organoid chip, Quintard et al. used cyclic olefin copolymer (COC) to fabricate a microfluidic device with U-cup-shaped trapping regions and serpentine microchannels. Although COC was not mechanically actuated, its optical clarity, dimensional stability, and precisely defined channel architecture enabled controlled organoid positioning, hydrogel deposition, air-assisted loading, and continuous perfusion. This design supported endothelial self-organization, vascular network formation, perfusable connections, and organoid maturation under flow (Quintard et al., 2024). This case clarifies how platform material and geometry can organise the mechanical and transport environment even when the material itself is not elastically deformed.

PDMS-based systems provide a more direct example of actively deformable platform materials. In a myogenic mechanobiology platform, Zhou et al. used PDMS as a mechanically responsive substrate. The elastic PDMS structure deformed under pneumatic loading, while embedded fluorescent beads mapped the resulting strain field and type I collagen coating improved myogenic cell adhesion on the culture surface. This design delivered cyclic strain to cardiomyocytes and skeletal muscle cells and quantified the local stress-strain distribution at the cell-substrate interface. It also revealed strain-dependent changes in myogenic cell alignment, polarization, and growth (Zhou et al., 2026). A vascular example is provided by Zhao et al., who developed a patient-specific aortic dissection on-chip model using a PDMS-based microfluidic device. The PDMS platform contained a 500 µm main chamber connected to 80 µm inlet and outlet channels, providing a perfusable and mechanically defined environment for vascular cells. A three-dimensional porous polycaprolactone (PCL) scaffold was incorporated to better mimic the vascular cellular microenvironment. Under controlled perfusion, this platform transmitted wall shear stress-related mechanical cues to vascular cells. It helped reveal that elevated shear stress promoted endothelial dysfunction and smooth muscle phenotypic switching. The model also linked abnormal mechanotransduction with vascular remodelling mediated by the mechanosensitive ion channel PIEZO1 (Zhao X. et al., 2026).

Taken as a whole, this evidence suggests that mechanically responsive platform materials are valuable because they translate device-level forces or flow conditions into tissue-level mechanical cues. Future work should report deformation fields, recovery after repeated actuation, material drift, and the relationship between designed strain and cell-experienced strain.

### 2.2 Photo- and thermo-responsive platform materials

Photo- and thermo-responsive platform materials are device-forming materials whose structure or function can be controlled by light or temperature (Shafique et al., 2024; Barwig et al., 2024; Schmidleithner and Peham, 2024). In OoC systems, they are not mainly used as cell matrices or readout probes. Instead, they form part of the device architecture itself, such as patterned channels, local actuation regions, switchable gates, or flow-control surfaces. Their value lies in spatial control: light or heat can define where structures are formed, where pathways open or close, and where local stimulation occurs without changing the whole culture environment (Schmidleithner and Peham, 2024).

Representative photo- and thermo-responsive platform materials include photocurable polymers, PNIPAM-based thermoresponsive polymers, photothermal films, magnetic hyperthermia composites, and optothermal coatings. Across these systems, the key design requirement is spatially confined activation, so that light or temperature can define local geometry, flow access, permeability, or transport without disturbing the whole chip (Shafique et al., 2024; Barwig et al., 2024; Chen et al., 2024). Shafique et al. developed PLInk, a poly (ethylene glycol) diacrylate (PEGDA)-based photocurable ink for low-cost LCD 3D printing. PLInk acted as a photo-responsive platform material because it polymerised only in illuminated regions under 405 nm light. This local light-triggered polymerisation was used to fabricate embedded microchannels, thin membranes, membrane microvalves, micromixers, and OoC devices in a 384-well format. The PLInk-defined platform supported endothelial sprouting, cellular reorganisation, and migration over 5 days. This case indicates that photocurable platform materials can define channel layout, membrane thickness, compartmental separation, and tissue-tissue interaction (Shafique et al., 2024).

Barwig et al. reported a thermo-responsive route using poly (N-isopropylacrylamide) (pNIPAM) microactuators written directly inside microfluidic channels by two-photon direct laser writing. These pNIPAM structures remained swollen below their transition temperature and narrowed local flow gaps, but contracted above the lower critical solution temperature (LCST) to open the channel space, enabling particles or single cells to be trapped and released under flow (Barwig et al., 2024). Chen et al. further demonstrated optothermal control using a thin gold-film platform. Under local light irradiation, the gold film converted optical energy into heat and generated thermo-osmotic flow near the chip surface, allowing nanoparticles with different sizes, charges, and compositions to be trapped, sorted, and assembled in selected regions (Chen et al., 2024). Collectively, these reports indicate that photo- and thermo-responsive platform materials can define where structures are formed, where flow pathways open or close, and where transport is locally redirected within a microfluidic device.

More recent photo- and thermo-addressable microfluidic systems further show how remote stimuli may provide platform-level control. Parames-Estevez et al. developed a magnetic hyperthermia regulated microfluidic device, in which magnetic nanoparticles converted an alternating magnetic field into local heat to regulate temperature within the device (Parames-Estevez et al., 2025). He et al. reported stretchable on chip optical tweezers that used light driven manipulation to control particles in deformable microfluidic settings (He et al., 2026). These examples are discussed as OoC-related platform strategies rather than fully validated living OoC models because they demonstrate local heating, remote actuation, or particle manipulation in microfluidic systems but still require biological validation under long-term perfused culture. Therefore, platform material selection should be guided by the intended mode of control rather than by fabrication convenience alone: PDMS is suited to elastic actuation, COC provides dimensional stability and lower small-molecule absorption, photocurable resins enable complex printed geometries, and photo- or thermo-addressable materials provide remote spatial regulation but require careful assessment of optical dose, heat distribution, and leachables.

## 3 Stimuli-responsive membranes and adaptive biointerfaces

In OoC barrier models, membranes are not merely structural dividers; they are the sites where transport, mechanics, and tissue-tissue signalling converge. This interface is continuously exposed to cyclic strain, shear stress, inflammatory mediators, oxidative stress, enzyme activity, and permeability changes (Doryab et al., 2024). These cues have motivated the development of stimuli-responsive membrane materials that function as adaptive tissue interfaces rather than static partitions. Their responsiveness can be considered from two complementary perspectives. Mechanically responsive membranes focus on force transmission and deformation at the tissue interface, especially when membrane mechanics influence epithelial, stromal, and endothelial organisation (Licciardello et al., 2024). Biochemically responsive and cell-remodellable membranes focus on permeability, surface chemistry, degradation, and matrix reorganisation in response to disease-associated or cell-derived cues, allowing transport and tissue maturation to be adjusted within membrane-based culture systems (Kim et al., 2024). From this perspective, OoC membranes should be treated as responsive biointerfaces that adapt to tissue-level changes, not simply as separators (Corral-Nájera et al., 2023).

### 3.1 Mechanically responsive membranes

In barrier-type OoC systems, mechanically responsive membranes are the deformable interfaces through which physical forces are transferred to cultured tissues. Their role differs from that of platform materials: they sit directly between tissue compartments and therefore influence transport, adhesion, and strain sensing at the same time. When vacuum, pressure, flow, or cyclic stretch is applied to the device, the membrane determines whether the epithelial-endothelial interface experiences a physiologically meaningful deformation or only a weakened mechanical input (Ugodnikov et al., 2025; Ugodnikov et al., 2024; Kaya and Yesil-Celiktas, 2024). For this reason, membrane mechanics become part of the biological model rather than a background engineering detail.

Recent studies have explored mechanically responsive membrane strategies that combine deformability, selective transport, and biological compatibility beyond stiff track etched inserts (Mitta et al., 2025; Doryab et al., 2024; Newton et al., 2025). For these membranes, pore size and transparency alone are insufficient. The material also needs to recover after repeated deformation, maintain stable transport, support cell attachment, and avoid fatigue during long term actuation. Newton et al. fabricated tunable matrix-derived membranes by forming thin ECM-based hydrogel layers across microfluidic slots. The membrane was prepared from collagen type I, basement membrane extract (BME), and elastin, and its mechanical behaviour was further tuned by ECM concentration, gel thickness, and BS3 crosslinking. By adjusting these parameters, the authors controlled membrane stiffness, thickness, and pressure-strain behaviour. This allowed hydrostatic pressure and mechanical strain to be applied independently or in combination, which is important for barriers that experience coupled mechanical cues *in vivo* (Newton et al., 2025).

Mitta et al. used electrospun medical-grade thermoplastic polyurethane (TPU) to construct a thin fibrous membrane for lung alveolar modelling. The membrane retained elasticity during cyclic uniaxial stretching at breathing-relevant frequencies and supported alveolar epithelial-like cells for at least 15 days. Under air-liquid interface culture, the cells formed an epithelial monolayer and expressed surfactant protein A, suggesting that the elastic fibrous membrane provided both mechanical and biological support (Mitta et al., 2025). She et al. developed a sac-like hydrogel membrane to reproduce curved tissue barriers on chips. The membrane was fabricated from di-acrylated Pluronic F127 (F127-DA), which formed a flexible hydrogel interface with good mechanical stability. In a lung-on-a-chip model, this sac-like membrane withstood rhythmic mechanical stretching and fluid flow for at least 24 h, while supporting epithelial and endothelial culture across an alveolar-capillary-like barrier. This study illustrates how mechanically responsive membranes can move beyond flat porous inserts by combining curved geometry, stretchability, transport, and barrier formation in one cell-supporting interface (She et al., 2023).

These studies indicate that mechanically responsive membranes are most useful when they make deformation, transport, and barrier readout interpretable within one interface. Nevertheless, durability and attribution remain difficult: repeated strain can alter membrane mechanics or permeability, while cells may simultaneously remodel junctions and matrix coatings. Addressing this problem will require fatigue testing, permeability measurements during actuation, adhesion stability assessment, and controls that separate material deformation from true biological barrier disruption.

### 3.2 Biochemical and cell mediated remodellable membranes

In barrier type OoC systems, biochemical and cell mediated remodellable membranes are interfaces whose transport, adhesion, or matrix properties can be shaped by biochemical cues and cellular activity. Their role differs from mechanically responsive membranes. Instead of mainly transmitting strain, they help reproduce barrier changes associated with inflammation, altered matrix composition, enzyme activity, or cell matrix remodelling. This is important because barrier dysfunction *in vivo* often involves both cell junction disruption and extracellular matrix reorganisation, rather than a simple increase in pore based transport (Zhou et al., 2025).

Recent studies have therefore focused on remodellable membrane interfaces with tunable biochemical presentation, including functionalised polymer membranes, ECM derived membranes, and matrix derived interfaces permissive to cell matrix interaction (Sobejano de la Merced et al., 2025; Corral-Nájera et al., 2023; Newton et al., 2024). In these designs, selective transport needs to be coupled with stable adhesion cues, cell recognition, adjustable permeability, and epithelial endothelial communication during culture. These material strategies are reflected in several recent studies. Newton et al. developed ultrathin matrix derived membranes directly inside thermoplastic OoC devices by forming thin ECM sheets from type I collagen alone or from collagen mixed with basement membrane extract (BME). Because these membranes were assembled *in situ* over microfluidic channels, their thickness and permeability could be adjusted by changing ECM concentration and precursor gel thickness, while the resulting interface provided cell adhesive and basement membrane like cues for epithelial and endothelial culture (Newton et al., 2024). Sobejano de la Merced et al. took a surface functionalisation approach by modifying polyethylene terephthalate (PETE) membranes through controlled hydrolysis and EDC/NHS activation. This introduced reactive carboxyl groups for more stable attachment of coating proteins, including collagen type I, gelatin, poly L lysine, serum proteins, and Matrigel, improving A549 epithelial and EA.hy926 endothelial cell attachment while preserving membrane morphology and mechanical integrity (Sobejano de la Merced et al., 2025). Another recent example is the biomimetic ultrathin membrane used by Mou et al. in a human kidney glomerulus-on-chip. The membrane was fabricated from electrospun silk fibroin, which provided a thin, biocompatible, and ECM-like fibrous interface between epithelial and endothelial compartments. Compared with conventional PDMS or polycarbonate inserts, this membrane supported closer tissue-tissue communication and better reproduced glomerular barrier structure. In the kidney chip, it enabled podocyte-endothelial organisation, selective molecular filtration, fenestrated endothelial differentiation, and disease-relevant barrier dysfunction modelling (Mou et al., 2024).

Overall, biochemical and cell mediated remodellable membranes can improve barrier interfaces by providing stable adhesion cues, adjustable permeability, tunable mechanics, and a more permissive environment for cell matrix interaction. Nevertheless, distinguishing material effects from cell responses remains a central challenge for this class of membranes. Changes in transport may arise from membrane chemistry, ECM remodelling, altered ligand presentation, or true cell junction disruption. Resolving this issue will require membrane characterisation to be combined with transepithelial electrical resistance (TEER), tracer transport, live imaging, junctional markers, and inflammatory readouts.

Across membrane strategies, the material choice should be tied to the dominant barrier question. Elastomeric and TPU membranes are most useful for strain transfer, ECM-derived membranes improve biochemical presentation and tissue proximity, and functionalised polymer membranes improve adhesion on otherwise bioinert supports. This distinction helps separate material-mediated effects from cell-junction disruption or culture-induced injury.

## 4 Stimuli-responsive hydrogel matrices

Conventional hydrogel matrices have been widely used in OoC systems to provide hydrated, cell-compatible three-dimensional microenvironments. Natural ECM-derived gels, synthetic hydrogels, decellularised ECM-based gels, and hybrid networks support cell encapsulation, adhesion, migration, molecular diffusion, and tissue organisation by defining matrix stiffness, porosity, ligand presentation, and degradability (Kutluk et al., 2023; Shou et al., 2023). These materials have enabled organoid, tumour, vascular, cardiac, and fibrosis-related OoC models, but many remain relatively static once culture begins. Their mechanical and biochemical properties can be tuned before use, yet they do not always reproduce matrix stiffening, degradation, soluble-factor release, or cell-driven remodelling during disease progression or tissue maturation. This limitation has driven the development of stimuli-responsive hydrogel matrices, whose properties can change in response to physical, chemical, or biological cues. Their value lies in turning the OoC matrix from a fixed scaffold into a dynamic microenvironment for modelling tumour invasion, fibrosis, inflammation, vascularisation, and organoid maturation (Tevlek and Çetin, 2024).

### 4.1 Physicochemically responsive hydrogel networks

Physicochemically responsive hydrogel networks are matrices whose swelling, stiffness, mesh size, degradation, or release behaviour changes in response to local chemical or physical cues (Protsak and Morozov, 2025; Toews et al., 2025). In OoC systems, their value depends on whether the material response matches the biological process being modelled. A fast pH-triggered swelling or release response may be useful for acidic tumour niches, while slower enzyme-mediated degradation may better represent invasion, matrix remodelling, or tissue maturation (Gao and Xu, 2025). Photoresponsive networks add another layer of control by allowing matrix mechanics or degradability to be changed at selected positions during culture (Luo et al., 2024).

Studies on pH responsive, enzyme responsive, and photoresponsive hydrogel networks show how matrix transport, degradation, release, and local mechanics can be adjusted during culture (Cao et al., 2023; Soliman et al., 2024; Zauchner et al., 2024). For these materials, cytocompatibility and stiffness are only part of the design problem; response kinetics, spatial control, diffusion behaviour, and stability under perfusion also need to match the biological process being modelled.

Gupta et al. developed a pH responsive hydrogel on chip system for renal tumour modelling using sodium carboxymethyl cellulose/poly (vinyl alcohol)/agarose loaded with 5 fluorouracil. The hydrogel was integrated into a poly (methyl methacrylate) (PMMA) microfluidic chip with A498 renal carcinoma cells and showed pH dependent, diffusion controlled drug release under acidic tumour like conditions. This linked tumour acidity, matrix transport, sustained drug exposure, and apoptosis related responses within the same hydrogel supported chip model (Gupta et al., 2026). Zauchner et al. developed a matrix metalloproteinase (MMP)-degradable microporous poly (ethylene glycol) (PEG) hydrogel using four arm PEG vinyl sulfone, hyaluronic acid, dextran, arginine-glycine-aspartic acid (RGD) adhesive peptides, and an MMP sensitive peptide crosslinker. Polymerisation induced phase separation generated interconnected pores of about 5–20 µm. Human mesenchymal stromal cells and osteoblasts encapsulated in this matrix spread rapidly, formed three dimensional networks within 24 h, maintained these networks over long term culture, and deposited ECM proteins such as collagen. The material was also integrated into a perfused microfluidic chip, showing that enzyme responsive hydrogel networks can support cell driven matrix remodelling under dynamic culture conditions (Zauchner et al., 2024). Palmieri et al. reported a photoresponsive hyaluronic acid (HA) hydrogel based on strained alkyne modified HA and a photosensitive azide crosslinker. Light exposure allowed the hydrogel to be softened or degraded after cell encapsulation. In three dimensional culture, non irradiated gels kept cells mostly rounded, whereas light softened regions promoted cell spreading and primitive capillary like network formation. This demonstrates how photoresponsive crosslinks can regulate local matrix mechanics and cell self organisation in space and time (Palmieri et al., 2025).

Physicochemically responsive hydrogel networks are therefore useful when matrix transport, degradation, stiffness, or release needs to change during culture. Still, calibration remains difficult because pH, enzyme activity, light exposure, flow, and cell remodelling can overlap, making it hard to assign a biological response to a single material trigger. Addressing this problem will require response kinetics, diffusion profiles, degradation products, light dose, enzyme sensitivity, and matrix stability to be reported under perfused culture conditions.

### 4.2 ECM mimicking and cell mediated remodelling hydrogel matrices

ECM mimicking and cell mediated remodelling matrices differ from physicochemically responsive gels because their main trigger is cellular activity. Instead of responding mainly to externally imposed pH, light, temperature, or oxidative cues, these matrices are reorganised by adhesion, traction, protease secretion, matrix deposition, and cell migration (Naba, 2024). This distinction matters in OoC systems because many tissue behaviours occur within a three dimensional matrix volume. Vascular sprouting, tumour expansion, immune infiltration, fibrosis, and organoid maturation all require cells to reshape their surroundings rather than simply cross a planar interface (Li et al., 2024; Zhao G. et al., 2026).

Work on ECM mimicking and cell mediated remodelling matrices has focused on combining biochemical recognition, permissive mechanics, and controlled degradability (Kutluk et al., 2023; Song et al., 2025). For these matrices, stiffness and cytocompatibility are not sufficient on their own. The material also needs to present relevant ligands, relax or degrade at an appropriate rate, support matrix deposition, and allow tissue maturation over time. Sun et al. developed a dynamic gelatin/beta-cyclodextrin hydrogel to regulate human blood vessel organoids. The material was formed through host-guest interactions between gelatin and acryloylated beta-cyclodextrin, creating an injectable viscoelastic network with rapid stress relaxation. Blood vessel organoids encapsulated in this dynamic matrix showed stronger angiogenesis and greater differentiation into arteriole-like structures than those cultured in non-dynamic GelMA hydrogels. Mechanistically, the viscoelastic matrix promoted vascular patterning through Notch3-related signalling in mesenchymal cells and altered PDGFB expression in endothelial cells, showing that time-dependent matrix mechanics can direct organoid maturation rather than only support cell survival (Sun et al., 2025). Zhu et al. used a decellularised amniotic membrane ECM hydrogel enriched in collagen VI for induced pluripotent stem cell (iPSC)-derived islet organoids. This tissue-derived matrix improved organoid viability, functional maturation, and engraftment, while collagen VI was identified as a key ECM component supporting biomimetic islet architecture and physiological function (Zhu et al., 2025). A more recent example is the boronate ester based dynamic hydrogel developed by Neumann et al. for three dimensional organoid culture. Reversible boronate ester bonds created a network that could remodel over time while still maintaining structural support. This dynamic matrix allowed encapsulated organoids to expand and reorganise their surrounding microenvironment, showing that dynamic covalent chemistry can balance matrix stability with cell mediated reorganisation (Neumann et al., 2026).

These reports indicate that ECM mimicking and cell mediated remodelling hydrogels are most useful when tissue development depends on reciprocal cell matrix interaction. Reproducibility, however, remains a major challenge. Natural and decellularised matrices provide rich biological cues but may vary between batches, whereas synthetic or hybrid systems require careful tuning to preserve both controllability and biological relevance. Addressing this issue will require clearer reporting of ligand composition, degradation behaviour, stress relaxation, matrix deposition, batch variability, and long term organoid function under perfused OoC conditions.

The two hydrogel categories differ in how experimental control is achieved. Physicochemically responsive networks are preferable when a defined external cue, such as pH, enzyme activity, or light, needs to alter release, degradation, or local mechanics. ECM mimicking and cell mediated remodelling matrices are more appropriate when the biological question depends on reciprocal cell-matrix interaction. Synthetic hydrogels improve reproducibility, whereas natural and decellularised matrices provide richer biochemical cues but require stronger batch-to-batch characterisation.

## 5 Readout-oriented responsive materials

Readout-oriented responsive materials make dynamic biological changes measurable in OoC systems. Unlike responsive platform, membrane, or matrix materials, their main role is not to deliver stimuli or reshape the microenvironment, but to translate cellular activity into electrical, optical, electrochemical, or mechanical signals. This function is especially important for organoid-on-chip and long-term OoC platforms, where endpoint assays may miss transient changes during development, disease progression, or drug response (Brooks et al., 2023; Yang et al., 2024). Recent sensing-integrated OoC studies therefore emphasise electrical, optical, and mechanical monitoring as major routes for real-time and non-destructive analysis (Alghannam et al., 2025).

### 5.1 Electroactive and electrochemical readout materials

Electroactive and electrochemical readout materials are used when OoC systems need to follow biochemical secretion, ionic activity, impedance changes, field potentials, or redox signals during culture (Rezaei et al., 2025; Jin et al., 2020). Their role is different from that of stimulation or matrix materials: they translate biological activity into electrical or electrochemical outputs that can be recorded continuously. This is important in barrier, neural, intestinal, cardiac, and organoid on chip models, where functional changes may occur before visible morphological changes appear (Feng et al., 2026; Tian et al., 2024; Hong et al., 2025; Park et al., 2025).

Work in this area has focused on electrode and interface materials that improve charge transfer, molecular selectivity, tissue contact, and signal stability during perfused culture. These materials include noble metals, carbon-based conductors, stretchable metal or polymer composites, soft conductive interfaces, and enzyme-functionalised nanomaterials. Their performance depends on biocompatibility, antifouling behaviour, mechanical compliance, and calibration stability (Li and Zhao, 2025; Shi et al., 2025; Park et al., 2025). Tian et al. developed a drug evaluation platform based on non-destructive organoid fate-state monitoring using a graphene field-effect transistor. The graphene channel served as the electroactive sensing material, allowing changes in organoid state to be followed in real time without destroying the sample. This approach is useful for drug testing because it links material sensitivity with continuous monitoring of organoid responses (Tian et al., 2024). Hong et al. integrated a stretchable electrochemical interface based on gold nanotubes and polydimethylsiloxane (AuNTs/PDMS) into an intestinal epithelium chip. The Au nanotubes provided electrochemical activity for serotonin detection, while PDMS gave the interface stretchability during peristalsis-like deformation. Caco-2 epithelial cells were cultured on the AuNTs/PDMS surface, and the chip enabled real-time monitoring of fatty-acid-enhanced serotonin release under dynamic mechanical stimulation (Hong et al., 2025).

Selective biochemical sensing can also be achieved through enzyme-functionalised nanomaterials. Feng et al. developed an integrated neural disease-modelling platform for *in situ* glutamate monitoring using human induced pluripotent stem cell (hiPSC)-derived neurons and an MNP@ZIF-8/enzyme sensor. The sensing material combined conductive magnetic nanoparticles with porous ZIF-8, which increased the electroactive surface area and provided sites for enzyme immobilisation. The enzyme layer gave the sensor glutamate selectivity, allowing neurotransmitter release to be monitored directly during neuronal culture. Nanomaterial-enzyme interfaces can convert local biochemical activity into a real-time electrochemical readout in neural OoC-related models (Feng et al., 2026).

Electroactive and electrochemical readout materials are valuable because they improve the timing, sensitivity, and continuity of functional measurement in OoC systems. At the same time, several challenges remain, including electrode drift, protein fouling, enzyme instability, mechanical mismatch, and calibration under flow. Addressing these issues will require clearer reporting of baseline stability, sensing selectivity, response time, antifouling performance, and agreement with independent biochemical or electrophysiological assays.

### 5.2 Optical probe and photonic readout materials

Optical probe and photonic readout materials are used when OoC systems need spatially resolved, non-destructive monitoring without direct electrical contact. Their role is to convert local changes in pH, ion concentration, oxygenation, molecular binding, cell activity, or deformation into optical signals such as fluorescence, luminescence, spectral shifts, Raman signals, or structural colour changes (Sekhwama et al., 2024). Compared with electrochemical or mechanical readouts, optical and photonic materials are particularly useful for mapping where a signal arises within a tissue, matrix, or microchannel.

Material development in this area has focused on optical probes and photonic structures that improve spatial localisation, signal sensitivity, imaging depth, and compatibility with living cultures (Jacques et al., 2023; Zhang et al., 2024). These materials are useful only when their signals remain stable, background noise is low, light exposure does not damage the culture, and calibration remains reliable under perfusion or three-dimensional culture conditions (Spiteri et al., 2024). Direct laser written photonic crystal hydrogel sensors developed by Liu et al. provide an example of local optical sensing inside microfluidic devices. These sensors combined a swelling responsive hydrogel network with an ordered photonic lattice. When local pH or ionic strength changed, the hydrogel altered its swelling state, and the photonic lattice converted this change into a measurable wavelength shift at selected positions in the channel (Liu et al., 2024). An aptamer functionalised cholesteric liquid crystal photonic biosensor reported by Hussain et al. provides an example of molecule selective optical sensing. This biosensor was based on an interpenetrating polymer network film and incorporated microcystin recognition aptamers. The aptamer functionalisation enabled target recognition, while the cholesteric liquid crystal structure converted microcystin binding into concentration dependent optical shifts (Hussain et al., 2024).

Accordingly, the optical examples are described by evidence status: some are integrated with organoid or OoC-compatible culture systems, whereas others represent OoC-related optical materials that mainly demonstrate sensing chemistry, imaging depth, or photonic signal conversion.

Fluorescence based materials can monitor intracellular activity and cellular state more directly. Tirgar et al. used calcium sensitive fluorescent indicators in an imaging compatible cardiac organoid platform. The indicator converted intracellular calcium dynamics into fluorescence intensity changes, allowing calcium transients and drug related functional responses to be monitored during electrical pacing rather than inferred only from visible beating (Tirgar et al., 2025). Ma et al. developed organelle targeting two photon activatable fluorophores for living cell and tissue imaging. These probes contained targeting groups and photoactivatable fluorophore units, generating fluorescence after two photon excitation. This material design enabled deeper three dimensional visualisation of subcellular structures in liver, kidney, and heart tissues. Although this strategy is not yet a fully integrated OoC sensor, it offers a material route for monitoring cellular states inside thicker organoid on chip tissues without destructive sectioning (Ma et al., 2025).

Overall, optical probe and photonic readout materials are valuable because they preserve spatial information during live culture. Still, their use is limited by photobleaching, optical background, limited penetration depth, probe leakage, phototoxicity, and calibration under perfusion. Addressing these limitations will require clearer reporting of signal stability, light dose, imaging depth, spatial resolution, reversibility, and agreement with independent biochemical or electrophysiological measurements.

### 5.3 Mechanical and electromechanical readout materials

Mechanical and electromechanical readout materials are used to measure force, strain, stiffness, pressure, or contraction in OoC systems during culture (She et al., 2025; Nguyen et al., 2026). These materials are especially important in cardiac, skeletal muscle, vascular, and organoid on chip models, where morphology alone cannot fully indicate tissue function. Their value lies in detecting functional abnormalities that may remain invisible in static images, such as weak contraction, abnormal rhythm, altered stiffness, or unstable mechanical coupling (Yin et al., 2025). Their defining feature is deformation sensitive signal conversion: when living tissues stretch, compress, bend, or displace the sensing interface, the material changes its resistance, impedance, resonance, voltage output, or mechanical position (She et al., 2025; Yin et al., 2025; Nguyen et al., 2026).

Material development in this area has focused on readout interfaces that remain mechanically compatible with living tissues while producing stable signals during repeated contraction, perfusion, or long term culture (Sekhwama et al., 2024). Sensitivity is only useful when the material also matches tissue stiffness, adheres reliably, recovers after deformation, resists fatigue, and can be calibrated across the expected force range. A recent original study by Svěrák et al. provides a more direct example of biomechanical readout in cardiac organoid models. The system used a silicon nitride cantilever for atomic force microscopy (AFM) as the mechanical sensing material, with two beating human pluripotent stem cell (hPSC)-derived cardiac organoids connected by a multicellular bridge. During contraction, organoid motion produced vertical deflection and torsional bending of the cantilever, which converted contractile behaviour into measurable mechanical signals. This platform distinguished synchronized and desynchronized beating, detected focal and conductive arrhythmic responses, and supported drug-induced cardiotoxicity assessment (Svěrák et al., 2026). Yin et al. used a related electromechanical strategy for dynamically beating human cardiac organoids by coupling resistive skins with microelectrode arrays. The resistive skin captured contraction related mechanical signals, while the microelectrode array recorded electrical activity. This dual material interface allowed real time electro mechanical profiling of beating organoids, making it possible to link contraction strength and rhythm with electrophysiological behaviour in the same model (Yin et al., 2025). A more recent example is the wireless biomechanical well plate developed by Nguyen et al. for contactless electromechanical monitoring of cardiac organoid and three dimensional cardiac tissue contraction. This system combined piezoresistive silicon cantilevers with a microscale liquid membrane through air mediated coupling. During tissue contraction, this coupled structure converted liquid membrane deflection and small pressure changes into silicon cantilever bending and resistance changes. The contactless configuration enabled submillinewton contraction forces to be measured across multiple cardiac tissues without clamping the organoids or placing sensors directly in the culture medium. The same system also supported wireless recording, electrical stimulation studies, temperature testing, and drug related contraction analysis (Nguyen et al., 2026).

Mechanoresponsive and electromechanical readout materials are valuable because they convert subtle tissue generated deformation, contraction, or stiffness differences into quantitative signals. Even with this advantage, coupling remains a major challenge: organoid growth, matrix remodelling, repeated contraction, and perfusion can shift the contact between tissue and sensor. Addressing this issue will require sensor stiffness, working range, fatigue behaviour, contact stability, spatial alignment, and calibration against independent force or motion measurements to be reported more clearly.

Readout-oriented materials should be selected according to the biological signal of interest rather than sensitivity alone. Electrochemical interfaces are suited for soluble biomarkers and impedance shifts, optical probes preserve spatial information, and mechanical or electromechanical sensors quantify contraction, pressure, or stiffness. For all three routes, baseline drift, calibration range, tissue-material coupling, and agreement with independent assays should be reported.

## 6 Discussion

This review highlights how stimuli responsive materials are extending organ on a chip (OoC) systems beyond externally controlled microfluidic culture. Across platform materials, membrane biointerfaces, hydrogel matrices, and readout materials, the main value of responsiveness is not material novelty itself, but the ability to make dynamic tissue behaviour controllable, localised, and measurable under defined experimental conditions. By linking stimulus delivery, adaptive microenvironment formation, tissue interface regulation, and real time functional readout, these materials make OoC systems better suited for modelling barrier disruption, tumour invasion, fibrosis, vascular remodelling, organoid maturation, electrophysiological dysfunction, and drug induced stress (Tevlek and Çetin, 2024; Zhang et al., 2026; Rezaei et al., 2025).

Responsive materials can also create additional uncertainty. Although a material that changes during culture may improve biological realism, the same response can complicate data interpretation. For instance, platform materials may show uneven deformation in soft-rigid or bioprinted microfluidic structures (Aye et al., 2025), while photo- or thermo-actuated regions may generate unintended local thermal gradients during operation (Parames-Estevez et al., 2025; Schmidleithner and Peham, 2024); membrane biointerfaces may show altered permeability because of material swelling, changed strain transfer, tight junction opening, or cell injury (Mou et al., 2024; Ugodnikov et al., 2024); hydrogel matrices may soften because of intrinsic stress relaxation or active cell remodelling (Yang et al., 2025; Soliman et al., 2024); and readout materials may drift because of fouling, photobleaching, matrix swelling, or true biological adaptation (Martinelli et al., 2024; Spiteri et al., 2024). These problems become more pronounced in perfused OoC systems, where flow, cells, proteins, degradation products, and repeated stimulation interact over time. Without careful calibration, responsiveness may become a source of noise rather than a useful design feature.

A practical way to address this uncertainty is to report which part of the response is material-driven, which part is architecture-driven, and which part is cell-driven. For example, deformation maps, tracer permeability, live-cell viability, matrix rheology, and independent biochemical assays should be combined when interpreting whether a measured change reflects material behaviour or a true tissue response.

Importantly, the next stage of development will require responsive material behaviour to be reported in a more transparent and comparable way across OoC studies. Rather than simply stating that a material is responsive, future work needs to define how the response occurs under chip relevant conditions. Platform materials should be characterised by actuation range, deformation recovery, optical or thermal distribution, and long term compatibility (Schmidleithner et al., 2024). For membranes, strain transfer during actuation, permeability changes, pore stability, and adhesion durability should be reported, while porous membrane fabrication studies highlight the need for standardised assessment of membrane structure and transport reliability (Williams et al., 2025). Hydrogel matrix studies should report stiffness drift, degradation kinetics, swelling, stress relaxation, and cell mediated remodelling over time, with particular attention to matrix stability, diffusion behaviour, and microenvironmental consistency in dynamic OoC culture (Liu et al., 2025). For readout materials, baseline drift, fouling resistance, calibration range, signal specificity, and tissue material coupling need to be documented (Martinelli et al., 2024). Such reporting would make it easier to distinguish material response from cellular response and to compare results across laboratories.

To make such reporting more useful, minimum reporting parameters should be module-specific. For platform studies, key parameters should include actuation amplitude, recovery ratio, fatigue behaviour, temperature or light distribution, and bonding stability. Membrane studies should include pore geometry, permeability before and during actuation, strain transfer, coating durability, and barrier readout controls (Parames-Estevez et al., 2025; Doryab et al., 2024). Hydrogel studies should include stiffness drift, swelling ratio, degradation kinetics, stress relaxation, diffusion profiles, and batch variability. Readout studies should include baseline drift, calibration curve, response time, limit of detection where relevant, fouling resistance, and comparison with independent assays (Soliman et al., 2024; Rezaei et al., 2025).

Beyond material characterisation, practical implementation and standardised use will depend on how responsive components behave inside integrated OoC systems. As future devices combine platform actuation, adaptive membranes, dynamic matrices, and real time sensing, cross talk between components will need closer attention. Several potential problems may arise: matrix degradation products could interfere with sensor performance, membrane deformation may alter transport across compartments, and optical stimulation may produce unintended heating in neighbouring tissues, as suggested by studies on integrated hydrogel-electrode systems and photoactuated microfluidic devices (Martinelli et al., 2024; Schmidleithner et al., 2024). Hardware complexity is another possible barrier. High throughput organoid on chip platforms still require careful control of perfusion, parallel culture formats, and readout compatibility during scale up (Kong et al., 2026). Continuous electrical, optical, chemical, and mechanical monitoring can also generate large multimodal datasets that are difficult to interpret manually. These barriers suggest that material innovation should be paired with miniaturised hardware, automated control, and artificial intelligence (AI) assisted analysis (Bai and Su, 2026).

Future validation should therefore move beyond demonstrating that a material responds. It should test whether the response improves a defined tissue-level endpoint, such as barrier integrity, contractile force, electrophysiological activity, metabolic function, inflammatory response, or drug sensitivity, compared with a non-responsive or mechanically matched control (Stresser et al., 2024). The most urgent obstacles differ by material class. Platform materials need more reliable long-term actuation and better control of unintended thermal or mechanical gradients (Obeid et al., 2024). Membrane biointerfaces require stronger evidence linking membrane mechanics to permeability and tissue-tissue signalling (Mou et al., 2024). Hydrogel matrices need improved reproducibility and time-resolved monitoring of matrix remodelling (Song et al., 2025). Readout materials require stable calibration under perfusion and clearer separation between sensor drift and biological adaptation (Yin et al., 2025).

Looking forward, the field is likely to move toward multifunctional and feedback capable OoC systems. Responsive materials may be integrated with 3D bioprinting, organoid engineering, wireless sensing, and machine learning based control to create platforms that monitor tissue state and adjust culture conditions in real time. However, this vision will only be useful if material responses are stable, interpretable, and biologically meaningful.

In conclusion, stimuli responsive materials have strong potential to advance OoC systems, but their success will depend on disciplined integration rather than novelty alone. A multidisciplinary approach linking materials science, microengineering, cell biology, sensing technology, data science, and regulatory validation will be necessary to connect material responsiveness with tissue level outcomes.
